# A familial case of Birt–Hogg–Dubé syndrome complicated with various cancers

**DOI:** 10.1002/rcr2.549

**Published:** 2020-03-11

**Authors:** Yuki Goto, Kazunori Tobino, Miyuki Munechika, Yuki Yoshimatsu, Hiromi Ide, Kosuke Tsuruno

**Affiliations:** ^1^ Respiratory Medicine Iizuka Hospital Iizuka Japan

**Keywords:** Birt–Hogg–Dubé syndrome, bladder cancer, lung cancer, pneumothorax, renal cancer

## Abstract

An 89‐year‐old woman with small papules on her face presented to our hospital complaining of progressive dyspnoea. Chest computed tomography (CT) showed bilateral multiple lung cysts, a nodular opacity in the right lower lobe, and bilateral pleural effusion. She was diagnosed with adenocarcinoma. Her son, a 65‐year‐old man, also had bilateral basally located lung cysts and a past medical history of spontaneous pneumothorax. He had multiple papules on the face and neck, which were pathologically diagnosed as fibrofolliculomas. We considered these cases to be Birt–Hogg–Dubé syndrome (BHDS). *Folliculin* (*FLCN*) gene mutations that may be tumour suppressive are suspected to be causative of this syndrome. FLCN dysfunction might lead to the development of various types of tumours other than renal tumours.

## Graphical abstract

Birt–Hogg–Dubé syndrome (BHDS) is a rare autosomal dominant inherited disorder characterized by fibrofolliculomas, renal tumours, lung cysts, and pneumothorax. This report described a familial case of BHDS complicated with various types of cancer (i.e. bladder, ureter, and lung).

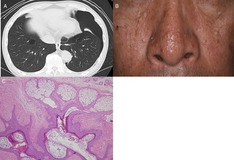

## Introduction

Birt–Hogg–Dubé syndrome (BHDS) is a rare autosomal dominant inherited disorder characterized by fibrofolliculomas, renal tumours, lung cysts, and pneumothorax 1. BHDS is caused by germline mutations in the *folliculin* (*FLCN*) gene, which encodes the folliculin tumour‐suppressor protein. It was reported that patients with this disease have a sevenfold higher risk of developing renal tumours 2; however, other various neoplasms have been described in patients with BHDS. We herein report a familial case of BHDS complicated with various cancers.

## Case Report

**Figure 1:**
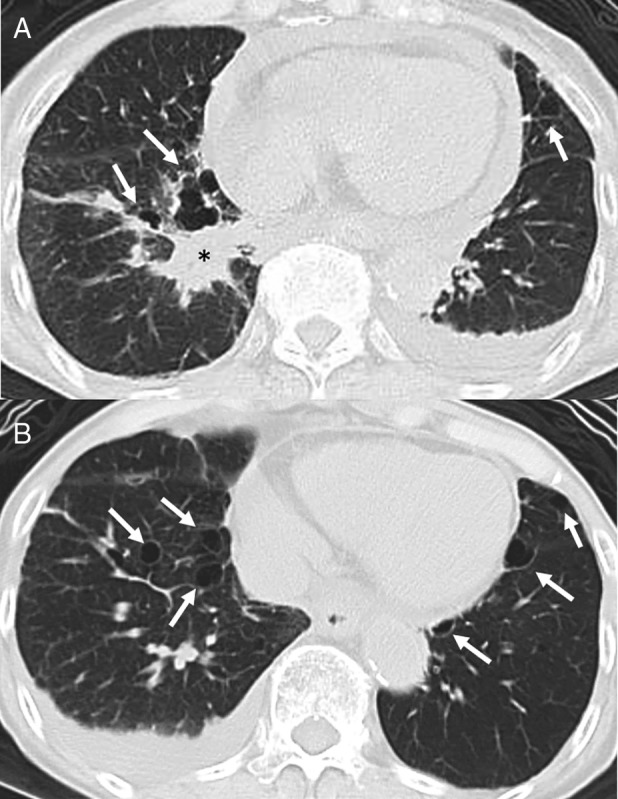
Chest computed tomography (CT) of the 89‐year‐old female patient (mother). Bilateral multiple lung cysts (arrows) were located in the basilar medial regions of the lung. A mass (*) was observed in the right lower lobe, with bilateral pleural effusion and pericardial effusion.

An 89‐year‐old woman was referred to our hospital complaining of progressive dyspnoea. She had a past medical history of bronchial asthma, gastric cancer, and bladder cancer. She had no history of smoking or exposure to any toxic materials. Her oxygen saturation on room air was 94% and her other vital signs were normal. On physical examination, small papules on and around her nose and cheeks were noted. Chest computed tomography (CT) scan demonstrated multiple cysts in both lungs (Fig. 1), which were predominantly located in the basilar medial regions of the lung. It also demonstrated a mass in the right lower lobe, bilateral pleural effusion, and pericardial effusion (Fig. 1). She underwent transbronchial tumour biopsy and a pathological examination revealed lung adenocarcinoma.

**Figure 2:**
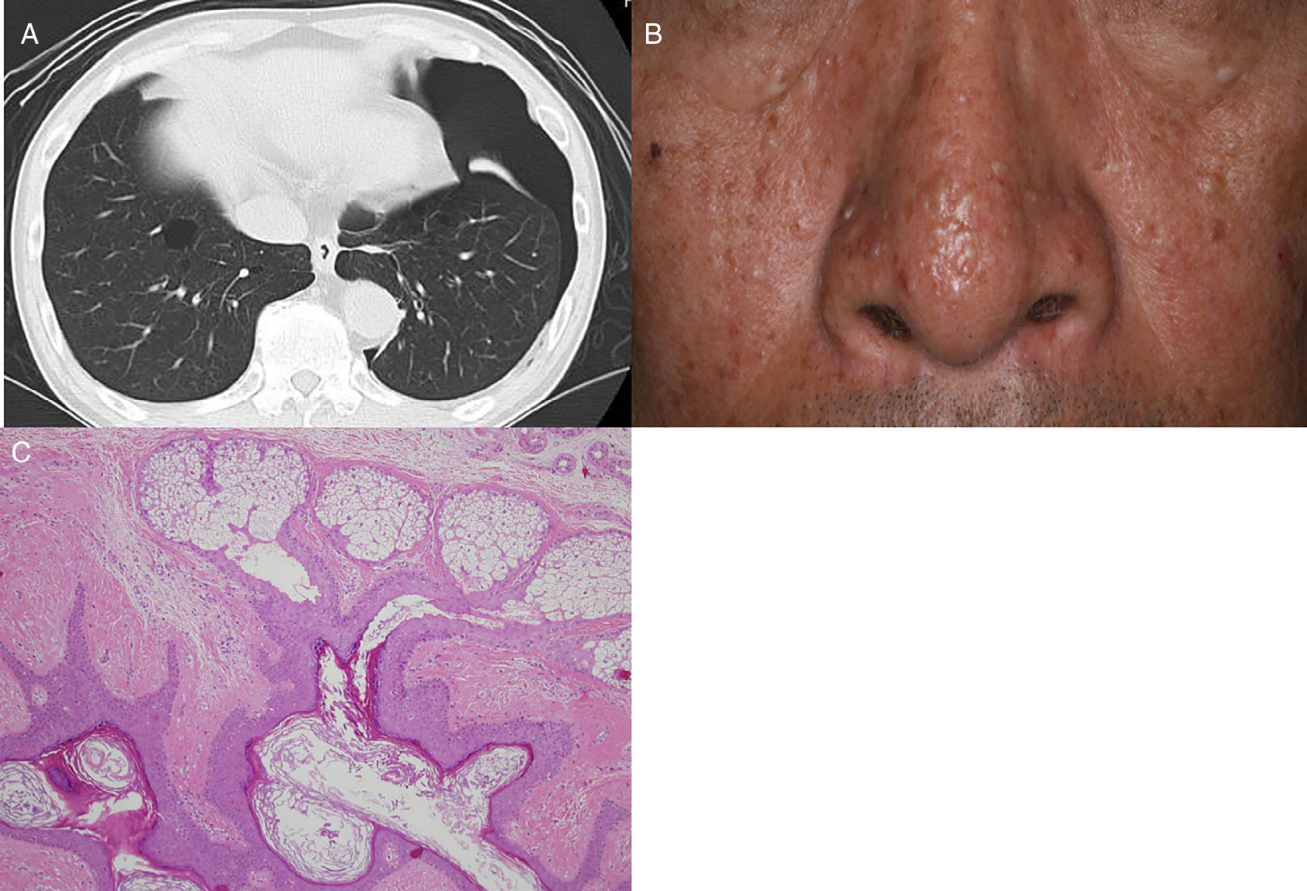
(A) Chest computed tomography (CT) of the 65‐year‐old male patient (son). Left‐sided pneumothorax and bilateral basally located multiple lung cysts were observed. (B) Multiple papules on the patient's face. (C) Pathological findings of a punch skin biopsy specimen from the patient's neck. A histological examination revealed dilated cystic follicles with thin epithelial strands emanating from the infundibulum into a dense collagenous stroma.

At the same time, her son, a 65‐year‐old man was referred to our department for pneumothorax. He had a past medical history of recurrent spontaneous pneumothorax, ureter cancer, and bladder cancer. He also had a 90 pack‐year smoking history. A physical examination revealed multiple papules on his face and neck. Chest CT demonstrated left‐sided pneumothorax and bilateral basally located multiple lung cysts (Fig. 2). He underwent punch skin biopsy and a pathological examination revealed fibrofolliculomas (Fig. 2). Although we did not perform genetic analysis, the patient met the diagnostic criteria for BHDS proposed by the BHD consortium: one major criterion, “at least five fibrofolliculomas or trichodiscomas, at least one histologically confirmed, of adult onset,” and one minor criterion, “multiple lung cysts: bilateral basally located lung cysts with no other apparent cause, with or without spontaneous primary pneumothorax” 1. His mother met two minor criteria (i.e. “typical lung cysts” and “a first‐degree relative with BHDS”) and was also diagnosed with BHDS.

The female patient decided not to receive anti‐tumour treatment because of her older age and low Eastern Cooperative Oncology Group Performance Status (3–4), and palliative care was started. She died of lung cancer at one and a half months after her admission. Her son's pneumothorax improved without treatment. Since then, he has been regularly monitored for recurrence of pneumothorax and urological tumours.

## Discussion

BHDS was first reported in 1977 and is an autosomal dominant inherited disorder caused by a germline mutation coding for *FLCN* mapped to chromosome 17p11.2, which encodes a highly conserved tumour‐suppressor protein. FLCN mRNA is expressed in various tissues, including the skin and its appendages, the distal nephron of the kidney, stromal cells and type 1 pneumocytes of the lung, epithelial ducts of the breast, acinar cells of the pancreas, serous parotid glands, and ovaries 3. Since *FLCN* gene was first reported in 2001, over 140 *FLCN* gene mutations have been reported. The most frequent of these is a hot spot mutation within a polycytosine C8 tract of exon 11; this has been found in the germline of 44% of BHDS patients 4. Its dysfunction might lead to the development of some types of tumours, including renal cancer. To date, several other tumour types have been reported in association with BHDS, including colon polyps and tumours, breast cancer, and lung cancer 5 Their real association with BHDS has not yet been clinically validated and additional studies are needed to address this issue.

With regard to our two patients, the former case (mother) had a past medical history of gastric and bladder cancer, and was diagnosed with primary lung adenocarcinoma at her most recent presentation. To the best of our knowledge, only four cases of lung cancer complicating BHDS have been reported (Table 1) 5, 6, 7. Three out of five reported patients (including our patient) were diagnosed with adenocarcinoma. *FLCN* mutations (c.1285dupC exon 11) were detected in two patients, whereas genetic analyses were not performed in the other two (including our patient).

**Table 1:** Previous reported cases of BHDS with lung malignancies.

Author	Age/gender	Smoking history	Clinical presentation	*FLCN* gene mutations
Nishida et al. 5	65/F	Current, 25 pack‐year	Adenocarcinoma	c.1285dupC exon 11
Nishida et al. 5	59/F	Current, 45 pack‐year	Adenocarcinoma	Not determined
Furuya et al. 6	Not determined/F	Not determined	Bronchoalveolar carcinoma	c.1285dupC exon 11
Gunji‐Niitsu et al. 7	38/F	Ex‐smoker, 0.6 pack‐year	Clear cell “sugar” tumour	c.1347_1353dupCCACCCT exon 12
This case (mother)	89/F	Never‐smoker	Adenocarcinoma	Not determined

BHDS, Birt–Hogg–Dubé syndrome; *FLCN*, folliculin.

On the basis of a functional analysis, it was reported that the *FLCN* gene may interact with FLCN‐interacting protein‐1 (FNIP1) and ‐2 (FNIP2), which induce signal transduction through 5′ AMP‐activated protein 1 (AMPK) and mammalian target of rapamycin (mTOR), while FNIP1 interacts with AMPK and regulates mTOR activity 8. The FLCN mutation may suppress or activate mTOR, the dysregulation of the mTOR pathway may result in the dysregulation of cell growth and protein synthesis, thereby leading to tumourigenesis 9. The patient's son had a past medical history of ureter and bladder cancer, both were histologically diagnosed as urothelial carcinoma. The causal relationship between BHDS and bladder cancer was suspected because the former case (mother) had no specific risk factors of bladder cancer; however, her son had the 90 pack‐year smoking history that is one of the risk factors of bladder cancer and little has been reported on the association between BHDS and bladder cancer.

In conclusion, *FLCN* mutations might be related to tumourigenesis in the lung or urinary organs. It is reported that most cancers developed after the appearance of fibrofolliculomas or pneumothorax. If BHDS can be diagnosed earlier based on these findings, the early detection and treatment of cancer may be possible.

### Disclosure Statement

Appropriate written informed consent was obtained for publication of this case report and accompanying images.
